# Use of Photo Elicitation Within Phenomenology: Depicting Lived Experience of Older Learners in an AFU

**DOI:** 10.1093/geroni/igaa057.1865

**Published:** 2020-12-16

**Authors:** Andrea Zakrajsek

**Affiliations:** Eastern Michigan University, Ypsilanti, Michigan, United States

## Abstract

While the use of photographs is an emerging data generation method within phenomenology (Plunkett, Leipert & Ray, 2013; Shulze, 2007), research that incorporates photo elicitation to inform the understanding of the lived experience is limited. This presentation will describe the use of photo elicitation within a phenomenological approach to explore the lived experience of older learners in higher education. After an initial interview, six participants aged 50 years and older shared photographs that that they chose to depict experiences of being student at a regional comprehensive university in the Midwest. Photographs served as mode for participant reflection of their experiences and guided a second interview. Interpretative Phenomenological Analysis (Smith et al., 2009) with photographs and verbatim transcripts ensued and resulting findings included: complicated sense of belonging and community development and access. Implications for use and analysis of photographs within qualitative research will be discussed.

